# Ceruloplasmin, Vitamin C, and Uric Acid Levels in Patients With Myocardial Infarction: A Comparative Cross-Sectional Study

**DOI:** 10.7759/cureus.56122

**Published:** 2024-03-13

**Authors:** Prajakta R Warjukar, Rina P Paunipagar, Dilip R Timalsina, Ankush V Mohabey, Pradeep B Jain, Swati P Panbude

**Affiliations:** 1 Biochemistry, Datta Meghe Medical College, Datta Meghe Institute of Higher Education and Research, Nagpur, IND; 2 Orthopedics, All India Institute Of Medical Sciences, Nagpur, IND; 3 Biochemistry, Jawaharlal Nehru Medical College, Datta Meghe Institute of Higher Education and Research, Wardha, IND

**Keywords:** oxidative stress, uric acid, vitamin c, ceruloplasmin, myocardial infarction

## Abstract

Introduction: Global mortality is significantly influenced by myocardial infarction. Scientists have examined the role of the copper-containing protein ceruloplasmin in heart attacks. It helps to regulate oxidative stress, iron metabolism, and inflammation. Vitamin C's antioxidative qualities lend credence to the idea that it could help prevent cardiovascular disease. Several studies have shown that elevated uric acid levels are related to a higher risk of myocardial infarction. With this background, we conducted this study to estimate levels of ceruloplasmin, vitamin C, and uric acid in patients with myocardial infarction.

Materials and methods: A tertiary care hospital in central India carried out this comparative cross-sectional study. The study was conducted between December 2022 and April 2023. Patients of any gender with newly diagnosed myocardial infarction who received admission to the intensive care unit and had ST-segment elevation of at least 2 mm in two or more consecutive electrocardiogram leads were included in the patient group. The control group consisted of individuals who did not exhibit any changes associated with myocardial infarction. Based on sex, age, and body mass index, the 75 control and 75 patients were matched. Ceruloplasmin, vitamin C, and uric acid were analyzed and compared.

Results: The uric acid levels among the patient group were 10.34 ± 3.23 mg/dL, and among the controls, they were 3.45 ± 1.12 mg/dL (p<0.001). The ceruloplasmin levels among the patient group were 64.34 ± 4.21 mg/dL, and among the controls, they were 29.23 ± 3.82 mg/dL (p<0.001). The vitamin C levels among the patient group were 13.80 ± 0.94 μmol/L, and among the controls, they were 45.62 ± 4.34 μmol/L (p<0.001).

Conclusion: The patients with myocardial infarction demonstrated significantly elevated levels of ceruloplasmin and uric acid, while their vitamin C levels were lower in comparison. It is crucial to comprehend the underlying mechanisms through which these parameters influence the development of myocardial infarction.

## Introduction

Myocardial infarction is a significant contributor to mortality on a global scale. Ischemic heart disease, encompassing myocardial infarction, was responsible for roughly 9.48 million fatalities in 2019, making up approximately 16.9% of global deaths [1,2]. Around the world, there are regional variations in the prevalence and incidence of myocardial infarction. For example, myocardial infarction rates are higher in developed nations than in developing nations, such as the United States, Western Europe, and Australia [3,4]. However, more people are suffering from myocardial infarction in many emerging nations because of aging populations, urbanization, and lifestyle changes. For example, in India, myocardial infarction is among the leading causes of death [5,6].

In various studies, an increase in uric acid level has been associated with an elevated risk of myocardial infarction. Increased incidence and severity of coronary artery disease (CAD), oxidative stress, endothelial dysfunction, and inflammation have all been associated with higher uric acid levels [7-9]. All these elements contribute to the beginning and advancement of a heart attack. Studies have investigated the potential of ascorbic acid to reduce the risk of heart disease and protect against heart attacks [10]. It acts as an antioxidant and assists in preventing oxidative stress, inflammation, and dysfunction of the blood vessels' lining. These factors are all associated with the occurrence and development of a heart attack [11-14].

Ceruloplasmin, a copper-containing protein, has been studied by researchers in heart attacks. It plays a role in controlling inflammation, iron metabolism, and oxidative stress [15]. According to specific research, lower ceruloplasmin levels may be related to an elevated risk of heart attacks, suggesting a protective effect against cardiovascular disease (CVD). However, more research is required to understand the underlying mechanisms and demonstrate a conclusive link between ceruloplasmin and heart attacks. We conducted this study to examine the levels of ceruloplasmin, vitamin C, and uric acid among heart attack patients while also considering age- and gender-matched people without heart attacks as controls [15].

## Materials and methods

A tertiary care hospital in central India carried out this comparative cross-sectional study. The study was conducted between December 2022 and April 2023. The Institutional Ethics Committee of Shalinitai Meghe Hospital and Research Centre approved the study (approval number: SMHRC/IEC/2022/12-15).

To detect a minimum difference of 15 units in the average blood parameter values between the groups, with a confidence interval of 95% and a power of 80%, a minimum sample size of 70 participants per group was determined. Consequently, 75 patients with myocardial infarction and 75 controls were included in this particular study. Eligible patients were matched based on age, gender, body mass index, and associated diseases with the control group. Table 1 shows the eligibility criteria for the present study.

**Table 1:** Eligibility criteria of the study

Inclusion criteria	Exclusion criteria
Patient group: Patients of any gender with newly diagnosed myocardial infarction, who received admission to the intensive care unit and had ST-segment elevation of at least 2 mm in two or more consecutive electrocardiogram leads, were included in the patient group.	Family history of heart disease
Control group: The control group consisted of individuals who did not exhibit any changes associated with myocardial infarction.	Myocardial infarction on treatment
	Pregnancy
	Gout
	Cardiomyopathy
	Lesch-Nyhan syndrome
	Malnutrition
	Unstable angina
	End-stage renal failure
	Hepatic failure
	Multiple sclerosis

In addition, the patient's medical records were carefully reviewed to identify any pre-existing conditions or ailments that could have impacted the study. The study did not include patients who met any of the exclusion criteria. Before participation, the study objectives were explained to eligible participants, and they were required to provide written informed consent or have their duly appointed representative do so on their behalf. Data collection involved interviews, questionnaires, examinations of patient medical records, and blood samples. Demographic data and general information were gathered through interviews and a questionnaire. The participant's blood pressure was also measured using the recommended technique by the World Health Organization. During this procedure, patients were given 15 minutes to relax before their blood pressure was measured twice while seated, using the right hand. The average of the two readings was used to determine the patient's blood pressure.

Sample collection and testing

A sterile needle and syringe collected venous blood samples (5 ml) from each subject's antecubital vein. The blood samples that were drawn were placed in sterile centrifuge tubes and allowed to coagulate. Afterward, the serum was separated from the clotted samples by centrifuging them at 3000 rpm for three minutes. The serum was then extracted using a micropipette and transferred into Eppendorf tubes. Following the blood collection, a biochemical analysis was conducted. Firstly, serum uric acid level was determined using the Uricase-Peroxidase kit method (Beckman Coulter Uricase Kit (reference range for uric acid: female 2.6-6.0 mg/dl, male 3.5-7.2 mg/dl). Then, a colorimetric approach, utilizing the Ceruloplasmin Colorimetric Activity Kit (Invitrogen by Thermo Fisher), was employed to measure the ceruloplasmin level (reference range for ceruloplasmin: 14 to 40 mg/dL). Finally, vitamin C was quantified using a competitive enzyme-linked immunosorbent assay (ELISA) technique, specifically the Qualichek Vitamin C ELISA kit (reference range for vitamin C: 23-114 μmol/L).

Statistical analysis

EPI Info version 7.2 (Centers for Disease Control and Prevention, Atlanta, Georgia) was used to collect, compile, and evaluate the data. Percentages were used to express the qualitative variables. The quantitative variables were classified and expressed as percentages or as mean and standard deviation percentages. The chi-square or Fisher exact test was used to assess the differences in the two proportions. The significance level was set at 0.05 for all analyses, which were conducted with two tails.

## Results

The comparison of demographic parameters considered in the study is shown in Table 2. Patients and controls were matched based on age, gender, body mass index, systolic blood pressure, and diastolic blood pressure. The age group in patients was 55.67 ± 7.82 years, whereas the age group in controls was 56.12 ± 8.2 years. No significant correlation was found between patients and controls when they were compared based on age (p=0.3321). The body mass index in patients was 25.81 ± 5.1 kg/m^2^ whereas the body mass index in controls was 26.56 ± 3.03 kg/m^2^. No significant correlation was found between patients and controls when they were compared based on body mass index (p=0.8729). There was a significant correlation found between the systolic blood pressure of patients (135.23 ± 11.28 mmHg) and controls (118.45 ± 7.38 mmHg) (p=0.0213). There was a significant correlation found between the diastolic blood pressure of patients (93.31 ± 6.77 mmHg) and controls (76.11 ± 6.43 mmHg) (p=0.0344).

**Table 2:** Demographic particulars among cases and controls SD: standard deviation

Variable	Patients		Control		p-value
	Mean	SD	Mean	SD	
Age (years)	55.67	7.82	56.12	8.2	0.3321
Height (cm)	162.31	8.23	164.56	9.88	0.1221
Weight (kg)	72.81	12.22	73.34	10.67	0.6782
Body mass index (kg/m^2^)	25.81	5.1	26.56	3.03	0.8729
Systolic blood pressure (mmHg)	135.23	11.28	118.45	7.34	0.0231
Diastolic blood pressure (mmHg)	93.31	6.77	76.11	6.43	0.0344

**Figure 1:**
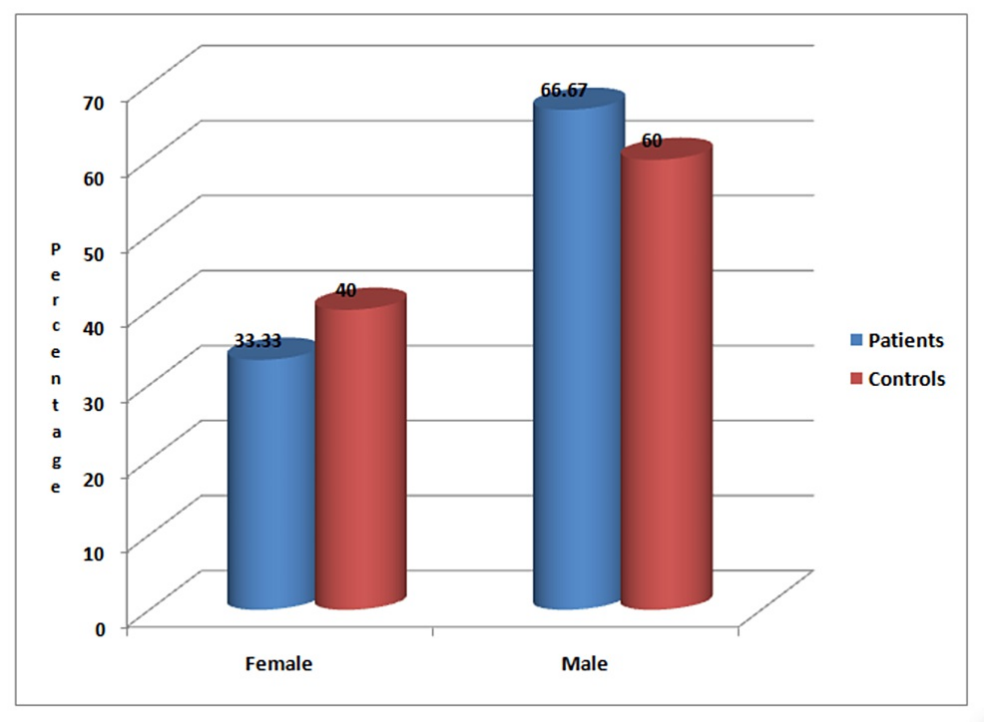
Gender-based distribution of the patients and controls

As shown in Figure 1, out of 75 patients studied, 30 were females and 45 were males, whereas out of 75 controls studied, 25 were females and 50 were males.

**Figure 2:**
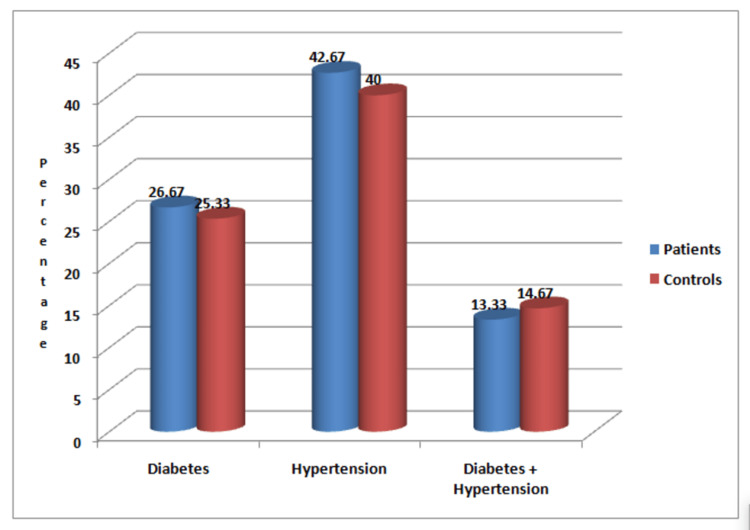
Distribution of the patients and controls based on associated diseases

Figure 2 shows the proportions of associated diseases (diabetes mellitus and hypertension) among the patient and control groups. The proportions of diabetes and hypertension were similar in patients and the control group (p>0.05).

Table 3 shows the comparison between the mean values of uric acid, ceruloplasmin, and vitamin C levels among the patients and the control group. The uric acid level among the patient group was 10.34 ± 3.23 mg/dL, and it was 3.45 ± 1.12 mg/dL among the control group (p<0.001). The ceruloplasmin levels among the patient group were 64.34 ± 4.21 mg/dL, and among the controls, they were 29.23 ± 3.82 mg/dL (p<0.001). The vitamin C levels among the patient group were 13.80 ± 0.94 μmol/L, and they were 45.62 ± 4.34 μmol/L among the controls (p<0.001).

**Table 3:** Comparison of mean values of uric acid, ceruloplasmin, and vitamin C levels among patients and controls SD: standard deviation

Parameter	Patients		Control		p-value
	Mean	SD	Mean	SD	
Uric acid (mg/dL)	10.34	3.23	3.45	1.12	<0.001
Ceruloplasmin (mg/dL)	64.34	4.21	29.23	3.82	<0.001
Vitamin C (µmol/L)	13.80	0.94	45.62	4.34	<0.001

## Discussion

Increasing evidence indicates that the excessive generation of reactive oxygen species (ROS) in chronic and acute pathological states significantly influences the progression of CVD [11,12,16]. A significant role is played by ROS in various signaling pathways that contribute to vascular inflammation and the development of atherosclerosis. In light of this, we investigated the levels of ceruloplasmin, vitamin C, and uric acid in the serum of patients with myocardial infarction.

The patients with myocardial infarction exhibited significantly elevated uric acid levels compared to the control group (p<0.001). A case-control study conducted by Mal et al. [17] found that, out of 200 people, 80 patients (about 40%) with acute myocardial infarction had hyperuricemia, which is characterized by very high uric acid levels in the bloodstream. The incidence of hyperuricemia in the control group (n=200) without acute myocardial infarction was 25% (n=50) (p<0.05). Similar findings were obtained in another study by Casiglia et al. [18], which indicated that 75% (n=188) of patients who suffered a fatal myocardial infarction (n=250) had elevated serum uric acid levels above the median cut-off value. Among the control group of 500 individuals without a history of fatal myocardial infarction, only 20% (n=100) had uric acid levels that surpassed the median cut-off value. Serum uric acid levels indicate oxidative stress and the activity of xanthine oxidase, serving as a practical measure in this regard. Uric acid can possess both pro-oxidant and antioxidant properties but may exacerbate endothelial dysfunction. In addition, uric acid can activate p38 MAP kinase, nuclear transcription factor NF-B, and AP-1, producing monocyte chemoattractant protein one. These chemokines are crucial in inducing vascular dysfunction and tissue damage, particularly following acute myocardial infarction events. This explains why uric acid is sometimes considered an inadequate marker to predict outcomes in cases of acute myocardial infarction.

The patients with myocardial infarction had considerably lower levels of vitamin C than the control group, according to the study's findings (p<0.001). Furthermore, the EPIC-Norfolk experiment showed that plasma vitamin C levels and the risk of heart failure, mortality from ischemic heart disease, and CVD are inversely related [19,20]. According to a study conducted by Nyyssonen et al. [21], called the Kuopio Ischaemic Heart Disease Risk Factor Study, there is a correlation between insufficient levels of vitamin C and an increased likelihood of developing ischemic heart disease. On the other hand, the Health Professionals Follow-Up Study by Rimm et al. [22] found no correlation between vitamin C intake and the incidence of coronary heart disease. According to the IOWA women's health research, supplemental vitamin C use was linked to a higher risk of CVD death [23]. The antioxidative properties of vitamin C support the possibility that it may be contributing to preventing CVD. In addition, several other effects of the vitamin also substantiate the idea that ascorbic acid can decrease the risk of CVD. For instance, it has been observed that vitamin C reduces the adherence of monocytes to the endothelium. The circulating monocytes adhere to endothelial cells, leading to the development of atherosclerotic plaques, and are considered an early indicator of atherosclerosis. Furthermore, it has been shown that vitamin C improves the endothelium's capacity to produce nitric oxide, promoting vasodilation and lowering blood pressure. Moreover, in cases where atherosclerosis is already present, ascorbic acid may delay vascular smooth muscle cell apoptosis, thereby promoting the excellent stability of plaques [13,14,24].

In the present study, the patient group's ceruloplasmin levels were considerably greater than those of the control group (p<0.001). According to Tang et al.'s [25] study, the highest quartile of serum ceruloplasmin levels had a greater risk of myocardial infarction than the bottom quartile, with a hazard ratio of 2.35 (95% CI 1.79-3.09). CAD's prevalence or incidence risk was not linked to genetic variations at the CP locus. In case-control research conducted by Kumar et al. [26], the CP levels were 61.8±3.8 for patients and 60.5±3.4 for controls; this difference was deemed significant (p<0.05). Ceruloplasmin possesses pro-oxidant properties. Together with H2O2, it generates hydroxyl radicals that contribute to DNA damage. This process may be facilitated by structural changes in ceruloplasmin, leading to the release of Cu2+ following oxidative degradation. During oxidative stress, the release of Cu2+ from ceruloplasmin enhances the production of free radicals, potentially worsening cellular damage. In CAD, ceruloplasmin levels rapidly increase after cellular injury, as it also acts as an acute-phase reactant protein.

There are certain limitations to the current study. Firstly, it was a cross-sectional study, and more precise results could have been obtained through case-control studies with larger sample sizes. Additionally, the current study did not explore the association of these parameters with disease progression. Therefore, longitudinal studies are needed to address this aspect. Finally, establishing real-time cut-off values for these biochemical parameters would aid clinicians in prognosticating patients. Nevertheless, this study represents one of the pioneering investigations conducted in our region, encompassing all three biochemical parameters.

## Conclusions

The research on individuals with myocardial infarction has revealed a significant rise in ceruloplasmin and uric acid levels, while their vitamin C levels tend to be lower. These findings underscore the importance of comprehending the mechanisms through which these substances contribute to the occurrence of myocardial infarction. Ceruloplasmin, an enzyme containing copper that participates in various bodily processes, and uric acid, a byproduct of purine metabolism, might have a role in developing myocardial infarction. Moreover, the reduced levels of vitamin C, a powerful antioxidant, could be linked to heightened oxidative stress and inflammation, both of which are associated with the progression of heart disease. Considering ceruloplasmin, uric acid, and vitamin C as potential indicators for myocardial infarction could provide valuable insights for future approaches to diagnosis, treatment, and prevention.
